# Association between Frailty and the Self-reported Inability to Immediately Open a Polyethylene Terephthalate Bottle Cap in Older Japanese Adults

**DOI:** 10.1298/ptr.E10323

**Published:** 2025-03-13

**Authors:** Yohei SAWAYA, Tamaki HIROSE, Masahiro ISHIZAKA, Naori HASHIMOTO, Akira KUBO, Tomohiko URANO

**Affiliations:** 1 Department of Physical Therapy, School of Health Sciences, International University of Health and Welfare, Japan; 2 Senior Services Division of Otawara, Otawara, Tochigi, Japan; 3 Department of Physical Therapy, School of Health Sciences at Odawara, International University of Health and Welfare, Japan; 4 Department of Geriatric Medicine, School of Medicine, International University of Health and Welfare, Japan

**Keywords:** Aged, Frailty, Japan, Polyethylene terephthalate, Screening

## Abstract

Objective: This study aimed to clarify the association between frailty and the self-reported inability to immediately open a polyethylene terephthalate (PET) bottle cap. Methods: This cross-sectional study included 427 participants aged 73 and 78 years in City A, Japan. Frailty was assessed using the Kihon Checklist (KCL), and PET bottle cap opening ability was surveyed using a mailed questionnaire. The participants were divided into Immediately Open and Cannot-Immediately Open groups. The frailty status and KCL scores were compared between the groups. Multivariate analysis was used to investigate the association between frailty and the PET bottle cap-opening pattern. The possibility of screening frailty based on the Immediately/Cannot-Immediately Open classification was analyzed using a receiver operating characteristic (ROC) curve. Results: The number of frailty in the Cannot-Immediately Open group was significantly greater than the expected count compared to the robust and pre-frailty groups (*P* <0.001). Total KCL scores differed significantly between the Immediately Open group (3.2 ± 3.1) and the Cannot-Immediately Open group (5.7 ± 4.1) (*P* <0.001). The Cannot-Immediately Open group showed significantly higher scores in 5 of the 7 domains and a higher proportion of 1-point scores in 15 of the 25 KCL items. Individuals in the Cannot-Immediately Open group were associated with frailty (β = 2.03; odds ratio = 7.62; *P* <0.001). The ROC analysis revealed a sensitivity and specificity of 50.0% and 81.0%, respectively (area under the curve = 0.65; *P* <0.001). Conclusions: The self-reported inability to open a PET bottle cap immediately may be a moderate screening tool for frailty and is associated with many frailty factors.

## Introduction

Frailty, characterized as “a state of diminished resilience to stress caused by an age-related decline in reserve capacity”^1)^, is a multifaceted concept encompassing physical, mental, psychological, and social dimensions^2–4)^. Frailty increases the risk of health problems, such as falls, disability, and death^5,6)^, and has been reported in longitudinal studies to increase the risk of various diseases, including dementia^7)^, diabetes^8)^, and cardiovascular diseases^9)^. Therefore, early detection and prevention of frailty are essential to promote healthy longevity in aging Japan. However, frailty assessment requires the use of questionnaires and interviews^1,10)^, which have limited ability to detect early signs of frailty. Therefore, it is important to identify daily activities performed by older adults that are associated with frailty.

To meet these requirements, we focused on the participants’ ability to open polyethylene terephthalate (PET) bottle caps. PET bottles are the most widely used beverage packaging material in the world^11)^. Previous reports have indicated that the ability to open a PET bottle cap reflects muscle strength and grip strength^12,13)^. This activity is considered a basic daily living skill^14)^ and a useful assessment tool^13,15)^. Among the items to be opened (PET bottle, snack, jelly, and pudding), the PET bottle cap requires the most force and a specific amount of force^16,17)^.

A previous study demonstrated an independent relationship between sarcopenia and the inability to open a PET bottle cap^18)^. It also demonstrated a required grip strength cutoff of 17.7 kg to determine whether an individual could open a PET bottle cap^18)^. Notably, this cutoff closely aligns with a cutoff of 18 kg for grip strength among females established by the Asian Working Group for Sarcopenia in 2019 and may serve as a valuable indicator of muscle weakness^19)^. They also devised a questionnaire to assess the subjective ability to open a PET bottle cap, and their findings indicated that this questionnaire could accurately discriminate grip strength^20)^. Other research groups in Japan have explored the connection between muscle weakness and cap-grasping patterns^21)^, highlighting the growing attention being paid in recent years to the ability to open PET bottle caps in daily life.

The relationship between the ability to open a PET bottle cap and sarcopenia or muscle strength has previously been elucidated. However, no studies have focused on the relationship between frailty, a crucial topic in the care of older adults, and an individual’s ability to open PET bottle caps. When physical function declines, psychological, mental, and social aspects may also weaken in a domino effect^22)^. Therefore, we hypothesized that there is also a connection between frailty, a multifaceted concept, and an individual’s ability to open PET bottle caps reflecting physical factors. This study aimed to clarify the relationship between frailty and the self-reported inability to immediately open a PET bottle cap, as assessed using the developed questionnaire.

## Methods

### Study design

This cross-sectional study was conducted between June and August 2023. All participants were given a written explanation of the questionnaire, and their completion of the survey was considered informed consent. The study was approved by the Ethics Review Committee of the International University of Health and Welfare (Approval numbers: 21-Io-38-2, 22-Io-25) and conducted in compliance with the Declaration of Helsinki.

### Study setting and participants

**Fig. 1.**
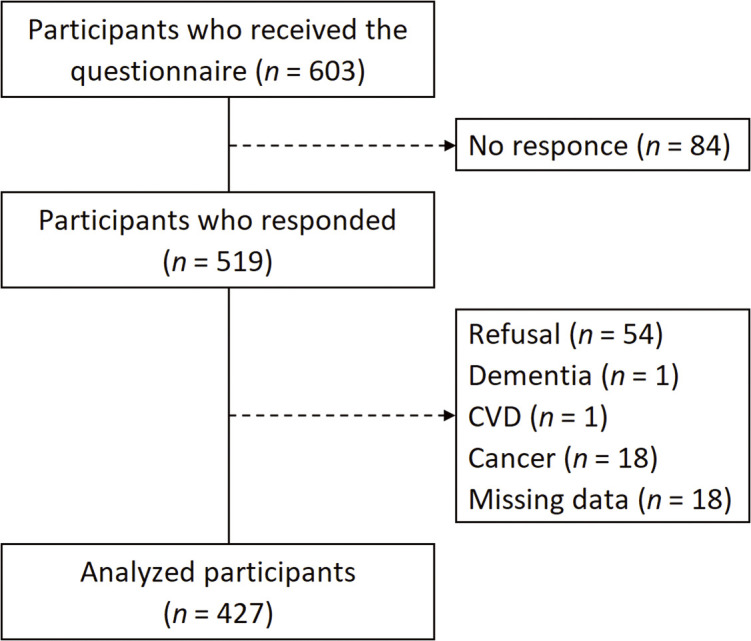
Flowchart of the participant enrollment process CVD, cerebrovascular disease

A questionnaire survey was conducted via mail. The targets were 603 individuals aged 73 and 78 years, excluding those certified as requiring long-term care in City A, Japan. The participants had participated in our previous study, which involved a complete survey focused on 2 age groups^23,24)^, and the data collected in 2023 constituted the new dataset. Therefore, the dataset used in this study did not overlap with data from any of our previously published articles. Those who died had new cases requiring long-term care or had moved to another city were excluded from the survey distribution list. Of 603 participants, 519 responded, yielding a response rate of 86.1%. Individuals who declined to participate, those with dementia, cerebrovascular disease, cancer, or missing data were excluded. Consequently, data from 427 participants (217 males and 210 females) were included in the analysis (Fig. 1). The analyzed data of all 427 participants had no missing values.

### Assessment of frailty

Frailty was evaluated using the Kihon Checklist (KCL), a self-reported questionnaire in which participants responded to each item with either “yes” or “no.”^25)^ This checklist consists of 25 items covering various aspects of daily life and is organized into 7 distinct domains^25)^. Each response was scored as either 0 (no impairment) or 1 (impairment), and the total score, ranging from 0 to 25, was obtained by adding the individual deficits. Higher scores corresponded to greater levels of functional decline. Based on previous studies, scores of 0–3 indicated a robust status, 4–7 indicated pre-frailty, and scores of 8 or higher indicated frailty^25,26)^. The KCL is reliable^27)^ and is recommended by both the Clinical Guide for Frailty in Japan and the Asia-Pacific Clinical Practice Guidelines^1,10)^. In addition, the physical domain of the KCL correlates with objective assessments such as the walking speed, Short Physical Performance Battery, 5-repetition chair stand test, and Timed Up and Go Test^28)^.

### Assessment of ability to open a PET bottle cap and study grouping

This study employed a questionnaire developed in a previous study to assess the participants’ subjective ability to open a PET bottle cap^20)^. The participants were then asked to recall and report their condition over the past month. This questionnaire assumes unopened PET bottles.

### PET Bottle Cap Opening Questionnaire

Question

Approximately how long does it take you to open the cap of a PET bottle?

Responses

(i) I can open it immediately.
(ii) If I repeatedly exert force, I can open it.
(iii) Sometimes I have to ask someone to open it for me.
(iv) I always have to ask someone to open it for me.

In this study, the participants who responded (i) above were categorized into the Immediately Open group, while those who responded (ii), (iii), and (iv) were categorized into the Cannot-Immediately Open group.

This questionnaire demonstrated high discriminatory validity, with an area under the curve (AUC) of 0.89 for handgrip strength when participants were grouped into (i) or (ii) and (iii) or (iv)^20)^. When classifying groups as can-open for (i) or (ii) and cannot-open for (iii) or (iv), the consistency with the actual ability to open a PET bottle showed a kappa coefficient of 0.77, indicating a significant actual agreement^20)^. According to this secondary data analysis, the percentage of people who could actually open the bottle cap for each of the 4 responses was (i) 99.0%, (ii) 87.9%, (iii) 36.4%, and (iv) 6.7%, with grip strength decreasing progressively: (i) 25.1 ± 7.0 kg, (ii) 21.5 ± 4.5 kg, (iii) 16.5 ± 3.5 kg, and (iv) 15.0 ± 3.5 kg^20)^. Therefore, although this questionnaire evaluates the subjective ability to open a PET bottle, it has demonstrated concurrent and convergent validity with objective indicators, such as the actual ability to open a PET bottle cap and grip strength.

### Other covariates

Weight, body mass index (BMI), sex, height, living arrangement (living alone or not), and medical conditions such as cerebrovascular disease, cancer, hypertension, and hyperlipidemia, were inquired through self-reported questionnaires.

### Statistical analyses

First, we compared the height, weight, and BMI of the Immediately Open and Cannot-Immediately Open groups using the Mann–Whitney U test, and age, sex, living alone status, and morbidity using the χ^2^ test or Fisher’s exact test. The Mann–Whitney U test was also applied to analyze total and domain-specific KCL scores, while the χ^2^ test or Fisher’s exact test was used to assess frailty status and the response distribution across the 25 questions. Additionally, the relationship between frailty and the ability to open a PET bottle cap was examined using binomial logistic regression analysis. The dependent variable was frailty status (Robust or Pre-frailty = 0, Frailty = 1), and the independent variable was the PET bottle cap-opening pattern. Two models were evaluated: Model I (unadjusted) and Model II (adjusted for age, sex, BMI <18.5, living alone, hypertension, and hyperlipidemia). A receiver operating characteristic (ROC) curve was generated to determine the sensitivity and specificity for screening frailty based on the opening patterns of the Immediately Open and Cannot-Immediately Open groups. Statistical analyses were performed using IBM SPSS version 25 (IBM Japan, Tokyo, Japan), with the significance level set at 5%.

## Results

### Participants’ basic attributes

The overall frailty status classification was robust in 247 (57.8%) participants, pre-frailty in 116 (27.2%), and frailty in 64 (15.0%). In the Immediately Open group, 326 participants answered (i), whereas in the Cannot-Immediately Open group, 101 participants answered (ii) 86, (iii) 14, and (iv) 1 to the questionnaire, assessing their ability to open a PET bottle cap. Table 1 compares the baseline characteristics of the participants in the Immediately Open and Cannot-Immediately Open groups. Significant differences were observed in age (*P* = 0.049), sex (*P* <0.001), height (*P* <0.001), and weight (*P* <0.001).

**Table 1.** Basic attributes and Kihon Checklist scores of the Immediately Open versus Cannot-Immediately Open groups

	Immediately Open group (*n* = 326)	Cannot-Immediately Open group (*n* = 101)	*P* value
Age			
73 years old	225 (69.0)	59 (58.4)	**0.049**
78 years old	101 (31.0)	42 (41.6)	
Sex			
Male	195 (59.8)	22 (21.8)	**<0.001**
Female	131 (40.2)	79 (78.2)	
Height (cm)	160.0 ± 8.8	154.5 ± 7.6	**<0.001**
Weight (kg)	59.8 ± 10.2	55.4 ± 9.5	**<0.001**
Body mass index (kg/m^2^)	23.3 ± 2.9	23.1 ± 3.2	0.680
Living alone	41 (12.6)	11 (10.9)	0.651
Morbidity			
Hypertension	157 (48.2)	48 (47.5)	0.911
Hyperlipidemia	53 (16.3)	22 (21.8)	0.202
KCL, 7 domains point			
Activities of daily living	0.4 ± 0.8	0.5 ± 0.8	0.644
Physical function	0.8 ± 1.0	1.6 ± 1.4	**<0.001**
Nutrition	0.2 ± 0.5	0.2 ± 0.5	0.908
Oral function	0.5 ± 0.8	0.9 ± 0.9	**<0.001**
Outdoor activity	0.2 ± 0.4	0.3 ± 0.5	**<0.001**
Cognitive function	0.3 ± 0.5	0.5 ± 0.7	**0.018**
Depression	0.7 ± 1.2	1.7 ± 1.7	**<0.001**
KCL, total point	3.2 ± 3.1	5.7 ± 4.1	**<0.001**
Frailty status			
Robust	211 (64.7)	36 (35.6)	**<0.001**
Pre-frailty	83 (25.5)	33 (32.7)	
Frailty	32 (9.8)	32 (31.7)	

The values given are mean ± standard deviation and number (%).

KCL, Kihon Checklist

### Groupwise comparison of KCL scores

The number of robust individuals in the Immediately Open group was significantly greater than the expected count compared to the pre-frailty and frailty groups. Additionally, the number of frailty individuals in the Cannot-Immediately Open group was significantly greater than the expected count compared to the robust and pre-frailty groups (*P* <0.001) (Table 1). The mean total KCL score differed significantly between the Immediately Open group (3.2 ± 3.1) and the Cannot-Immediately Open group (5.7 ± 4.1) (*P* <0.001) (Table 1). The Cannot-Immediately Open group showed significantly higher scores in 5 of the 7 domains of KCL scores: physical function (*P* <0.001), oral function (*P* <0.001), outdoor activity (*P* <0.001), cognitive function (*P* = 0.018), and depression (*P* < 0.001) (Table 1). Table 2 presents the intergroup comparison of the 25 questions in the KCL questionnaire. The Cannot-Immediately Open group exhibited a significantly higher percentage of impaired responses (1 point) for 15 of the 25 items, namely, activities of daily living (item 1), physical function (items 6, 7, 9, and 10), oral function (items 13, 14, and 15), outdoor activity (item 17), cognitive function (item 20), and depression (items 21–25).

**Table 2.** The proportion of responses to the 25-item Kihon Checklist for the Immediately Open versus Cannot-Immediately Open groups

No.	Questions	Answer = 1	Immediately Open group (*n* = 326)	Cannot-Immediately Open group (*n* = 101)	*P* value
1	Do you go out by bus or train by yourself?	No	15 (4.6)	10 (9.9)	**0.047**
2	Do you go shopping to buy daily necessities by yourself?	No	5 (1.5)	1 (1.0)	1.000
3	Do you manage your own deposits and savings at the bank?	No	28 (8.6)	4 (4.0)	0.123
4	Do you sometimes visit your friends?	No	69 (21.2)	19 (18.8)	0.609
5	Do your family or friends turn to you for advice?	No	25 (7.7)	14 (13.9)	0.059
6	Do you normally climb stairs without using a handrail or wall for support?	No	70 (21.5)	42 (41.6)	**<0.001**
7	Do you normally stand up from a chair without any aids?	No	23 (7.1)	30 (29.7)	**<0.001**
8	Do you normally walk continuously for 15 minutes?	No	40 (12.3)	19 (18.8)	0.096
9	Have you experienced a fall in the past year?	Yes	42 (12.9)	21 (20.8)	**0.050**
10	Do you have a fear of falling while walking?	Yes	88 (27.0)	45 (44.6)	**0.001**
11	Have you lost 2 kg or more in the past 6 months?	Yes	58 (17.8)	17 (16.8)	0.825
12	Height: cm, Weight: kg, BMI: kg/m^2^ If BMI is less than 18.5, this item is scored.	Yes	14 (4.3)	7 (6.9)	0.296
13	Do you have any difficulties eating tough foods compared to 6 months ago?	Yes	57 (17.5)	27 (26.7)	**0.041**
14	Have you choked on your tea or soup recently?	Yes	61 (18.7)	31 (30.7)	**0.010**
15	Do you often experience having a dry mouth?	Yes	61 (18.7)	36 (35.6)	**<0.001**
16	Do you go out at least once a week?	No	7 (2.1)	2 (2.0)	1.000
17	Do you go out less frequently compared to last year?	Yes	42 (12.9)	31 (30.7)	**<0.001**
18	Do your family or your friends point out your memory loss? e.g., “You ask the same question over and over again.”	Yes	32 (9.8)	15 (14.9)	0.158
19	Do you make a call by looking up phone numbers?	No	13 (4.0)	7 (6.9)	0.278
20	Do you find yourself not knowing today’s date?	Yes	45 (13.8)	25 (24.8)	**0.009**
21	In the last 2 weeks, have you felt a lack of fulfillment in your daily life?	Yes	22 (6.7)	27 (26.7)	**<0.001**
22	In the last 2 weeks, have you felt a lack of joy when doing the things you used to enjoy?	Yes	28 (8.6)	21 (20.8)	**0.001**
23	In the last 2 weeks, have you felt difficulty in doing what you could do easily before?	Yes	82 (25.2)	56 (55.4)	**<0.001**
24	In the last 2 weeks, have you felt helpless?	Yes	35 (10.7)	23 (22.8)	**0.002**
25	In the last 2 weeks, have you felt tired without a reason?	Yes	70 (21.5)	43 (42.6)	**<0.001**

Numbers are presented as number (%).

BMI, body mass index

### Association between frailty and cannot-immediately open grouping

Table 3 presents the results of the binomial logistic regression analysis exploring the factors associated with frailty. The inability to open a PET bottle cap immediately was associated with frailty in unadjusted Model I (β = 1.45; odds ratio = 4.26; 95% confidence interval [CI], 2.44–7.43; *P* <0.001) and adjusted Model II (β = 2.03; odds ratio = 7.62; 95% CI, 3.83–15.15; *P* <0.001).

**Table 3.** Association between frailty and polyethylene terephthalate bottle cap opening patterns using binomial logistic regression analysis

	β	Odds ratio	95% CI	*P* value
Model I				
Cannot-Immediately Open	1.45	4.26	2.44–7.43	**<0.001**
Model II				
Cannot-Immediately Open	2.03	7.62	3.83–15.15	**<0.001**

Dependent variables: Robust or Pre-frailty = 0, Frailty = 1.

Independent variables: Immediately Open group = 0, Cannot-Immediately Open group = 1.

Model I: Non-adjusted.

Model II: Adjusted for age, sex, BMI <18.5, living alone, hypertension, and hyperlipidemia.

BMI, body mass index; CI, confidence interval

### ROC curve analysis

**Fig. 2.**
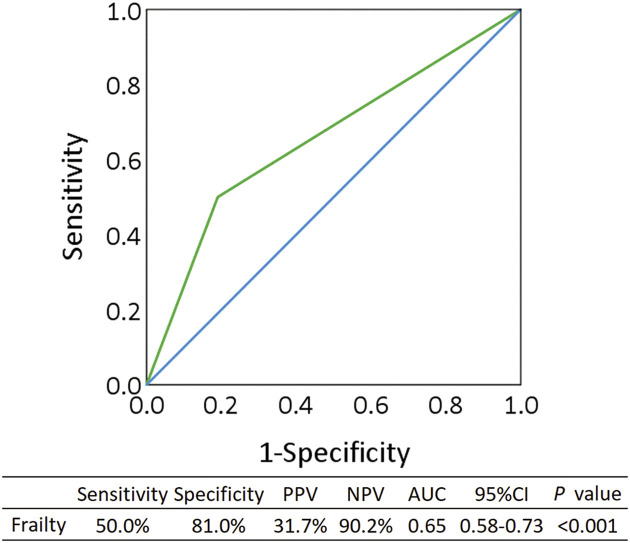
Screening ability for frailty based on the Immediately/Cannot-Immediately Open groups using a receiver operating characteristic curve AUC, area under the curve; CI, confidence interval; NPV, negative predictive value; PPV, positive predictive value

Figure 2 shows the ROC curve for screening frailty based on the Immediately Open/Cannot-Immediately Open classification. The sensitivity and specificity were 50.0% and 81.0%, respectively (area under the curve = 0.65; *P* <0.001).

### Supplementary analysis

The analysis targeting both the pre-frailty and frailty groups, conducted using the same methodology as in Table 3 and Figure 2, is presented in Supplementary Table 1 and Supplementary Figure 1.

## Discussion

To our knowledge, this is the first study to reveal an association between frailty and the participants’ ability to open a PET bottle cap. Most studies on the opening motion of PET bottles have focused on whether it is possible to open a bottle^12,16–18)^. The novelty of this study lies in its focus on whether it can be opened immediately, which is a slight weakness that people subjectively experience in daily life from middle to old age. Initially, the answers differed significantly between the Immediately Open and Cannot-Immediately Open groups for more than half of the KCL questions. Furthermore, the inability to open a PET bottle cap immediately was related to frailty in the multivariate analysis. Therefore, the subjectively perceived functional difference, whether or not immediately opening a PET bottle cap, was potentially associated with frailty.

These findings offer advantages in terms of generalization. First, the ability to open a PET bottle cap in this study was evaluated using only a single question. The methods can be applied to medical interviews conducted by healthcare professionals, such as physical therapists, as the questionnaire is designed to be straightforward and easy to answer. PET bottles are used worldwide for packaging beverages, and opening a bottle cap is a simple action that people of all ages, from children to older adults, perform in their daily lives. Slight difficulties encountered when opening a PET bottle cap can be noticed by older adults themselves or by their family and friends. This awareness can be utilized as a population strategy approach to frailty prevention and health promotion and is expected to contribute to the development of self-help and mutual assistance programs within the community.

This study conducted a group-wise comparison of the 25 questions of the KCL to determine whether the difference between the Immediately Open and Cannot-Immediately Open groups signified a specific component of frailty. Impairment rates were higher in the Cannot-Immediately Open group for 15 of the 25 items reflecting physical function and many other components. Focusing on physical function, significant differences were observed in questions no. 6, “climbing stairs without using handrail or wall for support,” and no. 7, “standing up from a chair without any aids,” suggesting an intergroup difference in muscle strength and balance abilities. Previous studies have demonstrated that the ability to open a PET bottle cap reflects muscle strength^12,13,18,20)^. Therefore, it is reasonable to observe impaired performance in these physical function items in the Cannot-Immediately Open group. Furthermore, significant differences were observed in 5 of the 7 domains in the intergroup comparisons. In the frailty domino effect, the physical function domino is considered the last domino^29)^. This means that if the physical function domino has fallen, it can be inferred that the preceding dominos, such as outdoor activity, depression, and oral function, had also fallen. Our findings suggest that categorizing individuals based on their subjective ability to immediately open a PET bottle cap reflects the multifaceted nature of frailty and encompasses not only physical frailty represented by muscle strength but also psychological, social, and oral aspects^2–4,30)^.

Additionally, screening for frailty through the self-reported inability to quickly open a PET bottle cap demonstrated moderate AUC values despite being based on a single question. A previous study screened for pre-frailty based on 9 factors classified among 25 predictors, with an AUC of 0.64, which is comparable to our result^31)^. Additionally, the predictive abilities of calf circumference, SARC-F, and SARC-CalF for sarcopenia, which are said to be associated with frailty, showed similar performances in terms of AUC, sensitivity, specificity, PPV and NPV as those in this study^32)^. An integrated interpretation of our results and those of previous studies suggests that it may be difficult to screen for frailty, a multifaceted factor, with high accuracy; therefore, further studies are needed.

This study had several limitations. First, the data were specific to 2 age groups within a single city. Additionally, the study participants were expected to be healthy because they were repeat participants. Because the survey was conducted via mail in collaboration with City A, detailed information on morbidity could not be obtained. Second, the number of participants needs to be increased for a stable analysis by sex. Third, the tightness of the PET bottle caps may vary depending on the product. However, previous research has shown that the ability to open PET bottle caps is consistent for 2 different products^20)^. Fourth, the evaluation of PET bottle opening ability in this study is based on the previous research and assesses subjective ability through self-report^20)^. A comprehensive study with a larger number of patients is required in the future.

## Conclusions

To our knowledge, this is the first study to elucidate the association between frailty and the ability to open PET bottle caps. These results indicate that the self-reported inability to immediately open a PET bottle cap is associated with the multifaceted nature of frailty and may be a moderate screening tool for frailty.

## Acknowledgments

We would like to thank all participants and staff involved in this study.

## Funding

This work was supported by JSPS KAKENHI Grant Numbers [22K17539 and 23K06873] and a JGS Grant for Geriatric Nutrition Research supported by Otsuka Pharmaceutical Factory, Inc.

## Conflicts of Interest

The researchers claim no conflict of interest.

## Supplementary Materials

Supplementary Table 1. Association between pre-frailty/frailty and polyethylene terephthalate bottle cap opening patterns using binomial logistic regression analysis.

Supplementary Figure 1. Screening ability for pre-frailty and frailty based on the Immediately/Cannot-Immediately Open groups using a receiver operating characteristic curve.
