# Supplementary material for: Association between Frailty and the Self-reported Inability to Immediately Open a Polyethylene Terephthalate Bottle Cap in Older Japanese Adults
Source: Phys Ther Res. 2025 Mar 13;28(1):37–44. doi: 10.1298/ptr.E10323 (PMC12047043; doi:10.1298/ptr.E10323)
Supplement: Supplementary Table 1. — Association between pre-frailty/frailty and polyethylene terephthalate bottle cap opening patterns using binomial logistic regression analysis. [file ptr-28-37-s01.pdf]

Supplementary Materials

Supplementary Table 1. Association between pre-frailty/frailty and polyethylene terephthalate bottle cap opening patterns using binomial logistic regression analysis

	ß	Odds ratio	95% CI	*P* value
Model I				
Cannot-Immediately Open	1.20	3.31	2.08–5.28	**<0.001**
Model II				
Cannot-Immediately Open	1.41	4.08	2.43–6.85	**<0.001**

Dependent variables: Robust=0, Pre-frailty or Frailty=1.

Independent variables: Immediately Open group=0, Cannot-Immediately Open group=1.

Model I: Non-adjusted.

Model II: Adjusted for age, sex, BMI<18.5, living alone, hypertension, and hyperlipidemia.

BMI, body mass index; CI, confidence interval.
