# Supplementary material for: Association between Frailty and the Self-reported Inability to Immediately Open a Polyethylene Terephthalate Bottle Cap in Older Japanese Adults
Source: Phys Ther Res. 2025 Mar 13;28(1):37–44. doi: 10.1298/ptr.E10323 (PMC12047043; doi:10.1298/ptr.E10323)
Supplement: Supplementary Figure 1. — Screening ability for pre-frailty and frailty based on the Immediately/Cannot-Immediately Open groups using a receiver operating characteristic curve. [file ptr-28-37-s02.pdf]

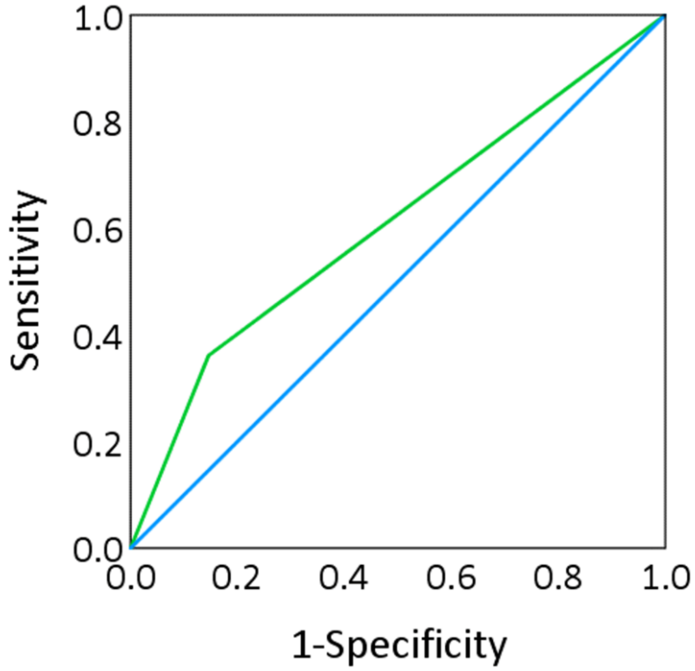

	Sensitivity	Specificity	PPV	NPV	AUC	95%CI	*P* value
Pre-frailty & frailty	36.1%	85.4%	64.4%	64.7%	0.61	0.55-0.66	<0.001

Supplementary Figure 1. Screening ability for pre-frailty and frailty based on the Immediately/Cannot-Immediately Open groups using a receiver operating characteristic curve

AUC, area under the curve; CI, confidence interval; NPV, negative predictive value; PPV, positive predictive value.
